# The *B. subtilis* MgtE Magnesium Transporter Can Functionally Compensate TRPM7-Deficiency in Vertebrate B-Cells

**DOI:** 10.1371/journal.pone.0044452

**Published:** 2012-09-06

**Authors:** Jaya Sahni, Yumei Song, Andrew M. Scharenberg

**Affiliations:** 1 Seattle Children’s Research Institute, Seattle, Washington, United States of America; 2 Department of Pediatrics and Immunology, University of Washington, Seattle, Washington, United States of America; Centre National de la Recherche Scientifique, Aix-Marseille Université, France

## Abstract

Recent studies have shown that the vertebrate magnesium transporters Solute carrier family 41, members 1 and 2 (SLC41A1, SLC41A2) and Magnesium transporter subtype 1 (MagT1) can endow vertebrate B-cells lacking the ion-channel kinase Transient receptor potential cation channel, subfamily M, member 7 (TRPM7) with a capacity to grow and proliferate. SLC41A1 and SLC41A2 display distant homology to the prokaryotic family of Mg^2+^ transporters, MgtE, first characterized in *Bacillus subtilis*. These sequence similarities prompted us to investigate whether MgtE could potentially compensate for the lack of TRPM7 in the vertebrate TRPM7-deficient DT40 B-cell model system. Here, we report that overexpression of MgtE is able to rescue the growth of TRPM7-KO DT40 B-cells. However, contrary to a previous report that describes regulation of MgtE channel gating by Mg^2+^ in a bacterial spheroplast model system, whole cell patch clamp analysis revealed no detectable current development in TRPM7-deficient cells expressing MgtE. In addition, we observed that MgtE expression is strongly downregulated at high magnesium concentrations, similar to what has been described for its vertebrate homolog, SLC41A1. We also show that the N-terminal cytoplasmic domain of MgtE is required for normal MgtE channel function, functionally confirming the predicted importance of this domain in regulation of MgtE-mediated Mg^2+^ entry. Overall, our findings show that consistent with its proposed function, Mg^2+^ uptake mediated by MgtE is able to restore cell growth and proliferation of TRPM7-deficient cells and supports the concept of functional homology between MgtE and its vertebrate homologs.

## Introduction

The divalent cation magnesium (Mg^2+^) is required for numerous biological processes and cellular functions in both prokaryotes and eukaryotes. Over the past decade, seven gene families have been suggested or identified as playing roles in magnesium homeostasis through molecular or genetic studies, including CorA, MgtA/B and MgtE [1], [2] in prokaryotes and TRPM6/7, SLC41A1/A2, MAGT1/TUSC3 and ACDP1-4 in eukaryotes [3]–[9]. Overexpression of MagT1 has recently been shown to partially rescue the growth impairment defect of TRPM7-KO DT40 B-cells [7] and mutations in the human *MAGT1* resulted in a novel X-linked immunodeficiency with magnesium defect, Epstein–Barr virus infection and neoplasia (XMEN) [10], [11]. Despite that, there is still a wide gap in knowledge between molecular or genetic identification of genes that modulate Mg^2+^ homeostasis, and functional characterization of the respective encoded proteins as well as the elucidation of mechanisms governing how their Mg^2+^ transport function is regulated.

While quite a few studies have shown functional substitution for the lack of either archaeal, prokaryotic or eukaryotic Mg^2+^ transporter by another prokaryotic/eukaryotic magnesium transporter [12], [13], [14], only a single report in yeast has demonstrated partial functional compensation of a eukaryotic transporter by a bacterial Mg^2+^ transporter, CorA [15]. SLC41 family of transporters are distant homologs of the bacterial MgtE proteins, and it has recently been shown that the human SLC41A1 is able to provide growth complementation in *Salmonella* strain MM281, which lacks any functional magnesium transporters [14]. Elucidation of the crystal structure of MgtE demonstrated that it comprises of two N-terminal cytoplasmic domains in addition to five transmembrane spans. Upon dimerization, the transmembrane domains form an ion-conducting pore that is highly selective for Mg^2+^ while the N-terminal cytoplasmic domains provide a regulatory activity that allows for Mg^2+^-dependent gating of the ion channel [16]. Furthermore, a conserved residue, D432, has been shown to be essential for magnesium-selectivity and transport activity of MgtE [17]. In a recent study, we showed that SLC41A1 can complement growth of vertebrate TRPM7-deficient (or knockout/KO) cells upon induction and that mutations of the corresponding pore residues in SLC41A1- D263 and D487, led to expression of non-functional transporters which exhibit normal surface trafficking [5]. These data suggested the existence of functional conservation between MgtE and SLC41A1.

Given the above results, we speculated whether MgtE could also provide functional substitution in TRPM7-KO cells. In the present study, we show that induction of MgtE expression in TRPM7-KO cells allows them to undergo proliferation in a manner analogous to what has been observed with SLC41A1 [5]. We further show that MgtE retains its membrane topology with its N-terminus localized in the cytoplasm, suggesting that it is likely capable of mediating trans-plasma membrane Mg^2+^ uptake in DT40 B-cells lacking TRPM7. Additionally, expression analysis of MgtE in the presence of 15 mM extracellular Mg^2+^ demonstrated that it exhibits magnesium-dependent downregulation, reflecting additional similarities with what has been previously observed with its distant homolog, SLC41A1 [5]. Finally, deletion of the cytoplasmic N domain of MgtE, whose precise function remains ambiguous, resulted in diminished cell growth and proliferation with cells displaying a strikingly smaller cell size. Collectively, our data demonstrates that MgtE mediates sufficient Mg^2+^ uptake in a heterologous vertebrate cell context to support robust proliferation and confirms a predicted regulatory role for its N-terminal cytoplasmic domain.

## Results

### Sequence Alignment and Cloning of the Prokaryotic MgtE in TRPM7-KO Cells

**Figure 1:**
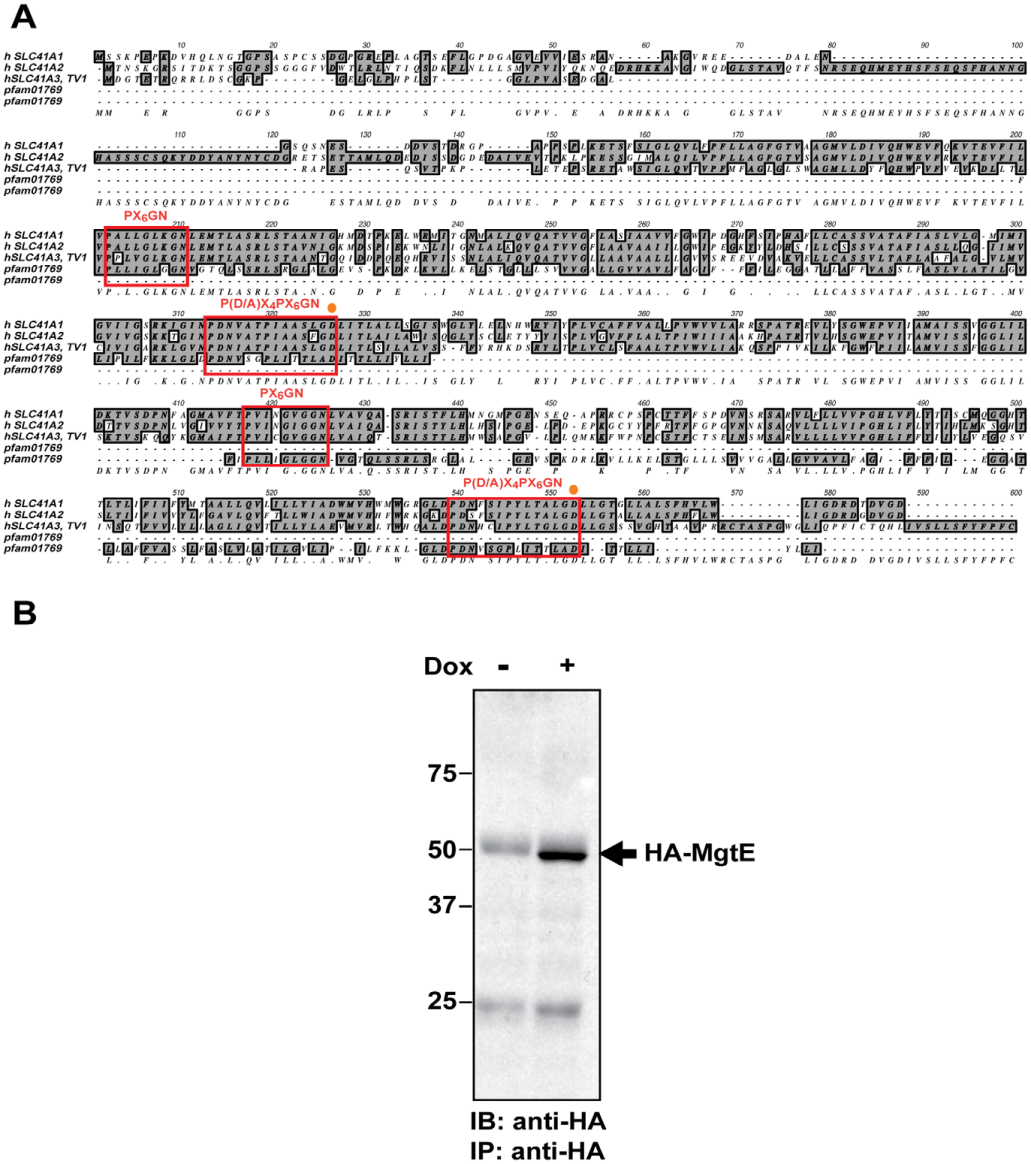
Sequence alignment of the human SLC41 transporter family with MgtE pfam 01769 and MgtE expression analysis. (**A**) ClustalW alignment of the amino acid sequences representing the three members of the human SLC41 transporters – A1, A2 and A3 transcript variant 1 (TV1) (includes both SLC41 D1 and D2 domains homologous to pfam01769) with the prokaryotic MgtE consensus sequence, pfam01769 from the conserved protein family database (pfam; http://pfam.sanger.ac.uk). The red boxes show the conserved motifs PX_6_GN and P (D/A)X_4_PX_6_D of which the former is considered to be the putative ion-conducting pore region and the last “D” (D432; aspartic acid residue) in the second motif has been established as an ion-selective site essential for Mg^2+^ uptake (denoted as an orange dot). (**B**) TRPM7-KO DT40 cells transfected with the N-terminal HA-tagged MgtE were doxycycline-induced and analyzed for MgtE protein expression by immunoprecipitation and immunoblotting with anti-HA. A clear band representing HA-MgtE was observed in the induced (dox +) cells. Data is representative of blots from at least two independent experiments.

Amino acid sequence alignments indicate that members of the eukaryotic solute carrier family 41 have substantial homology to the prokaryotic MgtE transporters (Figure 1A and [18]). In particular two conserved motifs - PX_6_GN and P(D/A)X_4_PX_6_D in the transmembrane region of MgtE are also present in the human SLC41 transporters, suggesting that MgtE and members of the SLC41 family are functionally homologous Mg^2+^ transporters. Further evidence of a functional homology between SLC41A1 and MgtE was recently provided by a mutational study, which showed that residues D263 and D487 of SLC41A1, corresponding to the last amino acid in the second conserved motif of MgtE, are essential for channel activity [5]. As SLC41A1 could complement the growth defect of TRPM7-KO cells, we asked whether a prokaryotic MgtE family member, whose function would be entirely orthologous to vertebrate cell physiology, would also be able to rescue the growth defect of TRPM7-KO cells in regular cell culture media. To answer this question, we generated a tagged version of MgtE by cloning its coding sequence in-frame with a haemagglutinin (HA)-tag at the amino-terminus. The construct was transfected into TRPM7-KO cells under the control of a doxcycline-inducible promoter, and a stable clone was analyzed for doxycycline-inducible expression of MgtE.

TRPM7-KO cells stably transfected with HA-MgtE were induced for 48 h with doxycycline and immunoprecipitation of the lysate was carried out by anti-HA followed by immunoblotting with the same antibody. A ∼51 kDa band corresponding to the predicted molecular weight of *Bacillus subtilis* MgtE was detected in the induced cells (Figure 1B). Additionally, we were also able to detect HA-tagged MgtE by direct immunoblotting, which suggests that it is likely expressed in high abundance in the cells (Figure S1A). Like a number of other membrane transporters [19], [20] including SLC41A1, MgtE displayed heat-induced aggregation. However, deletion of its N-terminal domain led to a significant reduction in its aggregation, suggesting that the amino acid residues in the N-domain of the protein in part might be responsible for the protein’s aggregative behavior (Figure S1B and S1C).

Taken together, our results demonstrate expression of MgtE in TRPM7-KO cells and show that addition of the HA-tag at its N-terminus does not interfere with its expression and assembly.

### Topology and Localization Analysis of HA-tagged MgtE in TRPM7-KO Cells

**Figure 2:**
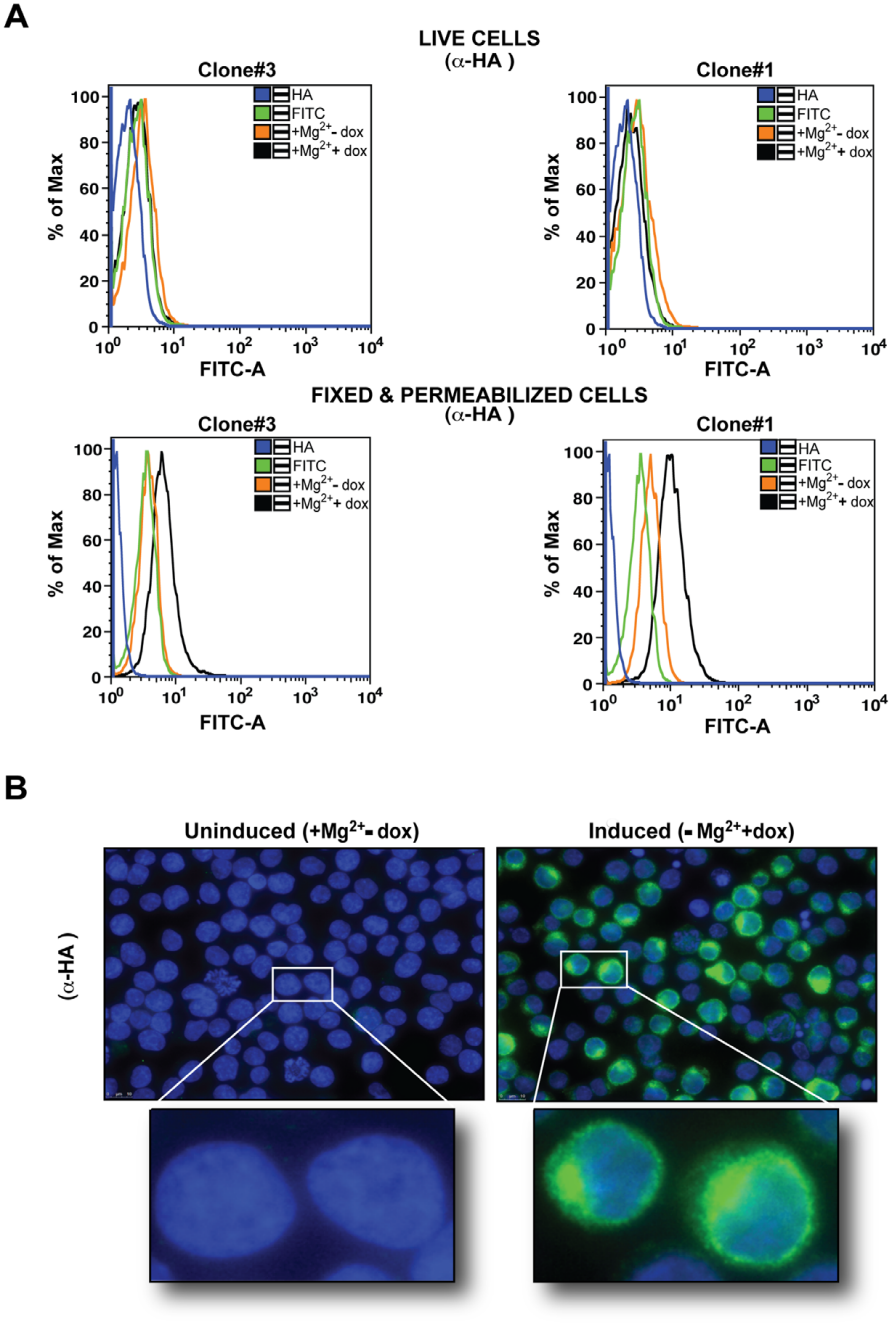
Membrane orientation and cellular localization analysis of HA-MgtE in TRPM7-KO cells. (**A**) Two clones of TRPM7-KO cells expressing HA-tagged MgtE (#3 and #1) were doxycycline-induced and either stained live with anti-HA (upper panel) or fixed & permeabilized and labeled with anti-HA (lower panel). Following staining with species-specific FITC, cells were acquired on BD LSRII. Data analysis by FlowJo demonstrated that the N-terminal HA-tag was only accessible intracellularly. (**B**) Both uninduced (left panel) and doxycycline inducible TRPM7-KO cells expressing HA-tagged MgtE (clone#3; right panel) were fixed & permeabilized followed by anti-HA and FITC staining for immunolocalization analysis of MgtE. The induced cells expressing the protein were clearly discernible from the uninduced cells on the basis of fluorescence. The upper panel shows staining of multiple cells in a single field and the lower panel shows a magnified view of two cells from the upper panel. All data are representative of results obtained from at least two independent experiments.

The crystal structure of *Thermus thermophilus* MgtE revealed that it forms a homodimer and consists of a cytosolic N-domain including a cystathionine beta-synthase (CBS) domain linked to five C-terminal transmembrane domains via a connecting helix [16]. In order to validate whether the predicted topology of MgtE is also preserved in vertebrate cells, we analyzed two clones (#3 and #1) induced with doxycycline in the presence of supplemental Mg^2+^, for expression of the HA-tagged protein by flow cytometry (Figure 2A). We reasoned that if MgtE were functioning as a trans-plasma magnesium transporter with an N-in/C-out topology, the N-terminal HA tag would not be detectable on the surface, but would be readily accessible intracellularly in fixed and permeabilized cells. Consistent with our hypothesis, we did not observe any significant shift in fluorescence with α-HA upon surface labeling of the induced cells compared to the uninduced controls (Figure 2A, top panel). However, in fixed and permeabilized cells, we observed a clear shift in the mean fluorescence of doxycycline-induced TRPM7-KO cells expressing MgtE, in contrast to the uninduced cells, confirming that the HA-tagged amino terminus of the protein is located in the cytoplasm. For all subsequent experiments and characterizations, clone #3 was used.

Next, to visualize the subcellular distribution of the overexpressed protein via indirect immunofluoresence, both uninduced and induced cells were fixed/permeabilized and stained with anti-HA followed by labeling with the species-specific anti-FITC (Fluorescein Isothiocyanate). The antibody staining was detected on the plasma membrane and the perinuclear region with punctate distribution throughout the cytoplasm suggesting that the HA-tagged MgtE also associates with the Golgi complex and endocytic compartments (Figure 2B, right upper and lower panels) [21], [22]. More importantly, MgtE-specific staining pattern was completely absent in the uninduced control cells (Figure 2B, left upper and lower panels).

Collectively, the above data indicates that despite expression in a heterologous background, MgtE is functional and retains its predicted topology in which its N-terminal region is in the cytoplasm.

### Growth Complementation and Analysis of Membrane Currents in MgtE-expressing Cells

Recently, Hattori M. et al demonstrated that expression of the wild-type (WT) MgtE is able to drive growth of a Mg^2+^ auxotrophic *E.coli* K12 strain lacking *corA, mgtA* and *yhiD* (*yhiD* encodes for the membrane protein related to MgtC), while an earlier study demonstrated that MgtE is also able to provide growth complementation in the mutant MM281 strain of *S.typhimurium,* which lacks any functional magnesium transport system and requires at least 100 mM extracellular Mg^2+^ for growth [17], [23]. A TRPM7-deficient vertebrate DT40 B-cell line, in which the ion-channel/protein kinase TRPM7 has been knocked out via gene targeting, was previously characterized in our lab. As TRPM7 plays an important role in magnesium homeostasis, these cells are only able to proliferate fully upon provision of 10–15 mM of supplemental Mg^2+^ in regular cell culture media [4]. In the past, others and we have also taken advantage of this cell line in order to characterize candidate vertebrate Mg^2+^ transporters and have shown that their inducible expression in the TRPM7-KO background is able to drive proliferation of TRPM7-deficient cells in regular cell culture media without provision of supplemental Mg^2+^ [5], [6], [7].

**Figure 3:**
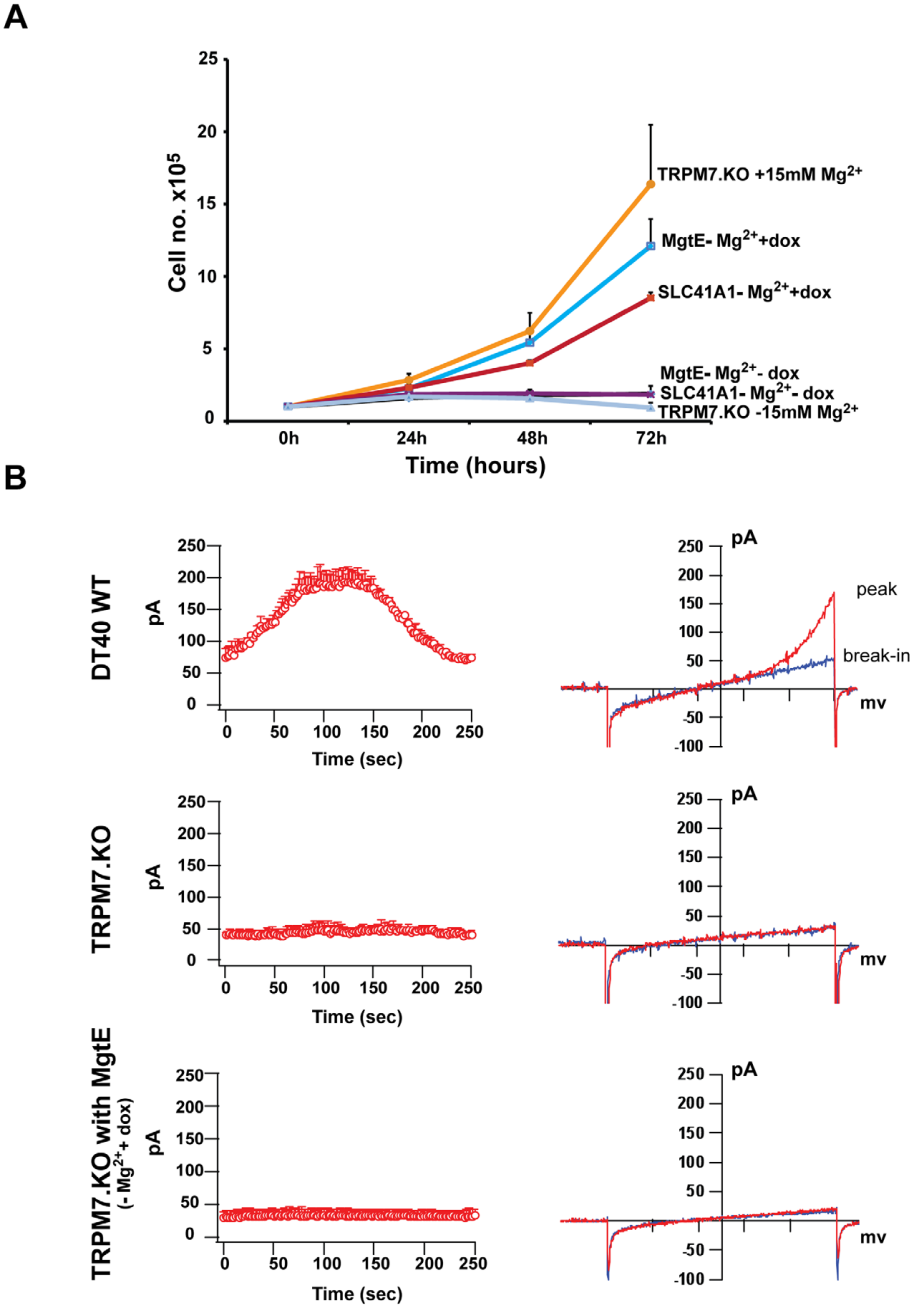
Growth and whole-cell patch clamp analysis of HA-tagged MgtE. (**A**) TRPM7-KO cells, TRPM7-KO cells overexpressing HA-tagged MgtE and TRPM7-KO cells overexpressing HA-SLC41A1-MYC were induced with doxycycline and their growth was analyzed. Growth curves represent the average of three independent experiments and the error bars represent mean ± standard error of the mean (SEM). TRPM7-KO cells with 15 mM supplemental Mg^2+^, TRPM7-KO cells inducibly expressing either tagged MgtE or SLC41A1 displayed growth complementation over a period of 72 hours while the uninduced cells and TRPM7-KO cells in regular cell culture media did not grow. (**B**) Whole cell patch clamp analysis of DT40 WT, TRPM7-KO and TRPM7-KO cells overexpressing MgtE. Left panel shows graphical representation of current development over time and right panel shows I/V curves at peak (in red) and break-in (in blue). Although a TRPM7 mediated MIC current was observed in DT40 WT cells, no MIC current was observed either in TRPM7-KO cells or TRPM7-KO cells expressing HA-MgtE.

To test whether a prokaryotic MgtE transporter could functionally provide TRPM7-KO cells with a capacity to grow and proliferate, TRPM7-KO cells expressing HA-tagged MgtE were doxycycline-induced and their growth was compared with both the parent TRPM7-KO cells as well as TRPM7-deficient cells overexpressing doxycycline-inducible human SLC41A1. As can be seen in Figure 3A, MgtE expressing TRPM7-KO cells proliferated nearly as well as the untransfected TRPM7-KO cells maintained in media with 15 mM supplemental Mg^2+^, while the uninduced cells and TRPM7-KO cells without supplemental Mg^2+^ did not proliferate. Interestingly, the proliferation capacity of MgtE-complemented cells was markedly higher than SLC41A1-complemented TRPM7-KO cells, indicating that MgtE could rescue the growth component of TRPM7-deficient cells at a level comparable with the vertebrate SLC41A1.

Hattori M. et al also reported MgtE-specific Mg^2+^-dependent currents in giant bacterial spheroplasts [17]. We sought to recapitulate their data and performed patch clamp experiments under the whole cell configuration in WT DT40 cells, TRPM7-deficient DT40 cells and TRPM7-deficient DT40 cells overexpressing MgtE (Figure 3B). The latter had been induced with doxycycline for at least 3–4 days prior to patch clamp analysis. In WT DT40 cells, we observed the development of typical MIC (Mg^2+^-inhibited cation current)/MagNUM (Mg^2+^-nucleotide-regulated metal-ion current) currents (Figure 3B, upper panel and [24]). These currents were completely absent in the TRPM7-KO DT40 cells, clearly demonstrating that the MIC/MagNUM currents were TRPM7-specific (Figure 3B, middle panel). However, under similar conditions we did not observe development of any MIC currents in TRPM7-deficient cells overexpressing MgtE, or any currents above the background levels in the TRPM7-KO DT40 cells (Figure 3B, lower panel), a surprising observation considering the fact that MgtE is expressed, folds properly in its heterologous environment and is able to support sufficient Mg^2+^ uptake to support cell growth.

Overall, despite our inability to detect MgtE-specific currents, our data shows that heterologous expression of a prokaryotic MgtE transporter is able to complement a Mg^2+^-dependent growth phenotype of TRPM7-KO cells, consistent with its functioning as a Mg^2+^ transporter capable of providing sufficient Mg^2+^ uptake to support a very rapid rate of cell growth.

### MgtE Expression and MgtE-mediated Growth are Downregulated at High Magnesium Concentration

Our SLC41A1 data revealed that the vertebrate transporter undergoes Mg^2+^-dependent post-transcriptional regulation with an obligate involvement of its N-terminal cytosolic domain in the process [5]. Based on this observation, and the demonstrated ability of MgtE to successfully complement cell growth, we contemplated whether the *B. subtilis* MgtE transporter might be subject to a similar regulatory mechanism.

**Figure 4:**
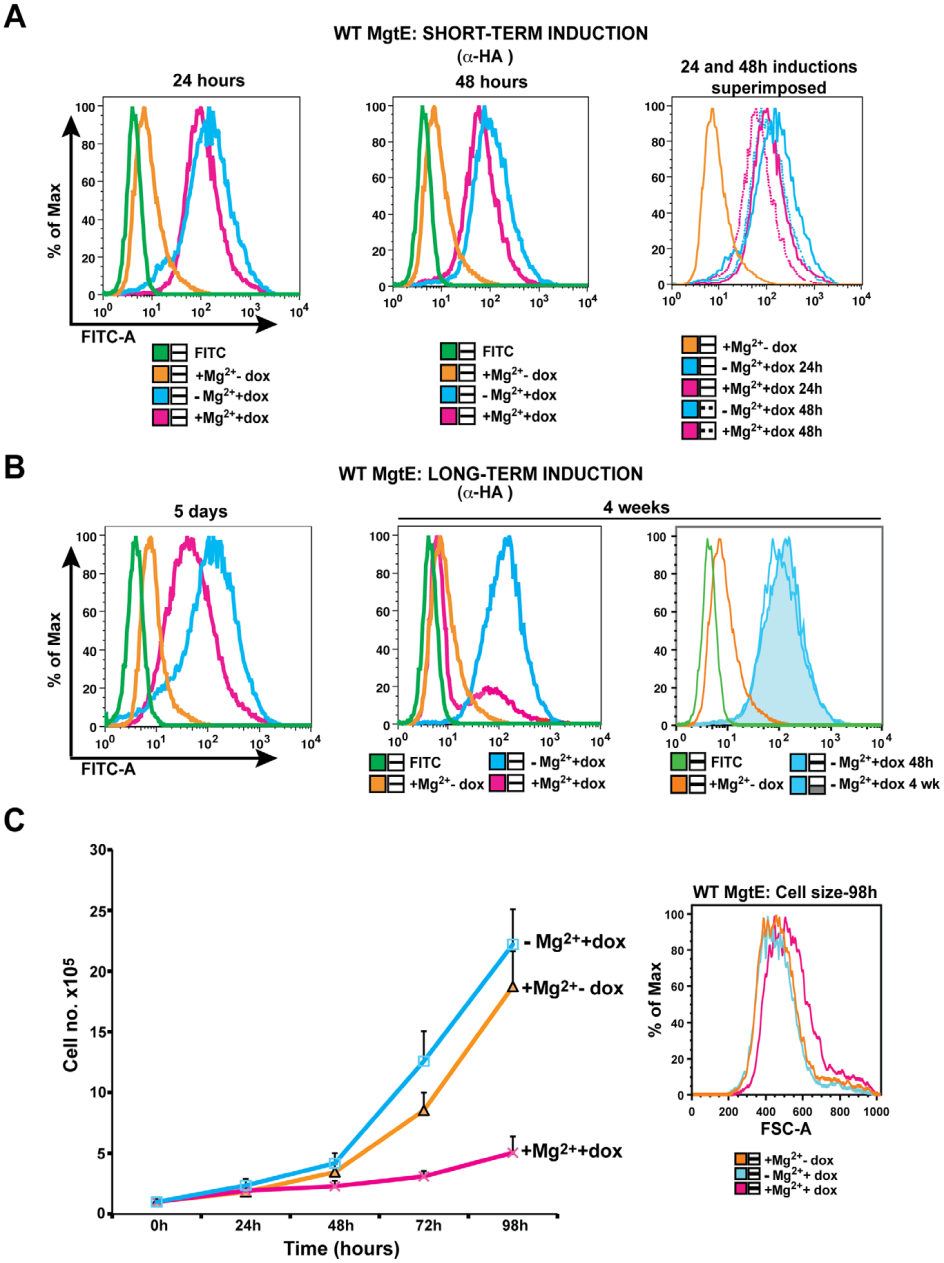
Downregulation of both MgtE expression and growth occurs in presence of high magnesium. (**A**) TRPM7-KO cells expressing HA-MgtE were induced either in presence or absence of 15 mM supplemental Mg^2+^ and analyzed for expression after staining with anti-HA and species-specific FITC on BD LSRII at 24 and 48 hours. Cells induced in regular cell culture media had higher MgtE expression compared to cells induced in presence of 15 mM Mg^2+^. (**B**) Cells from similar conditions as mentioned in (A) were analyzed for expression at 5 days and 4 weeks post-induction and displayed fluorescence shifts similar to what was observed in (A) with significantly lower MgtE expression in cells induced in presence of 15 mM Mg^2+^ at 4 weeks. All data are representative of results obtained from at least three independent experiments. (**C**) *Left panel*: TRPM7-KO cells expressing MgtE were induced either in presence or absence of 15 mM supplemental Mg^2+^ and their growth was determined for a period of 98 h. Growth curves represent the average of three independent experiments and the error bars correspond to mean ± SEM. Cells induced in presence of 15 mM Mg^2+^ exhibited diminished growth compared to cells induced in regular cell culture media. *Right panel*: Cell size analysis of samples in the left panel at 98 h. TRPM7-KO cells expressing MgtE were kept either uninduced or doxycycline-induced in absence/presence of 15 mM Mg^2+^ and acquired on BD LSRII. Cells induced in presence of 15 mM Mg^2+^ were bigger in size compared to the uninduced and induced cells. Results are representative of three independent experiments.

To test this, TRPM7-KO cells expressing HA-tagged MgtE were induced either with only doxycycline or doxycycline in presence of 15 mM supplemental Mg^2+^ for 24 and 48 hours and analyzed for total protein expression with anti-HA staining using flow cytometry. We found that MgtE expression was comparatively higher in doxycycline-induced cells than the cells induced in presence of supplemental Mg^2+^ at both 24 and 48 hours (Figure 4A). Interestingly, the data set in Figure 4A replicates to an extent what we have previously observed with SLC41A1, wherein expression of SLC41A1 was subject to Mg^2+^ dependent regulation [5]. For additional validation, we allowed cells in both the groups to continuously proliferate under similar conditions for a period ranging from five days up to four weeks and analyzed MgtE expression by flow cytometry (Figure 4B). At five days post induction, cells induced in presence of both doxycycline and 15 mM Mg^2+^ displayed a strikingly lower shift in fluorescence compared to cells that were only doxycycline induced (Figure 4B, left panel). Furthermore, induction under parallel conditions for a period of up to four weeks resulted in a dramatically lower fluorescence shift of MgtE expressing cells induced in presence of supplemental Mg^2+^ whereas TRPM7-KO cells expressing MgtE and induced with only doxycycline, showed stable, high levels of expression at both 48 hrs and 4 weeks post-induction (Figure 4B, right panel). We further assessed whether the Mg^2+^ mediated downregulation specifically pertains to MgtE and found that neither DT40 WT cells inducibly expressing the HA-tagged human pyruvate kinase M2 (PKM2) nor TRPM7-KO cells inducibly expressing the double-tagged SLC41A2 (FLAG-N terminus; HA-C terminus), a distant vertebrate homolog of MgtE, displayed any discernable shifts in their fluorescence intensity in presence of 15 mM Mg^2+^ (Figure S2).

To investigate how this magnesium-dependent downregulation of MgtE affects growth, we induced TRPM7-KO cells expressing MgtE either with/without 15 mM Mg^2+^ and analyzed their growth rates over a period of 98 hours (Figure 4C, left panel). Over this five-day period, MgtE-expressing cells induced in presence of 15 mM Mg^2+^ displayed significantly stunted growth along with reduced MgtE expression (Figure 4B), but had a larger cell size than uninduced cells and cells induced only with doxycycline (Figure 4C, right panel). This latter observation suggests that the combination of MgtE expression and supplemental Mg^2+^ might be resulting in Mg^2+^ overload, as we typically observe reduced cell size when TRPM7-deficient DT40 cells are subject to Mg^2+^ restriction. DNA content analysis of the uninduced (+Mg^2+^-dox), induced (-Mg^2+^+dox) and cells induced in presence of 15 mM Mg^2+^ (+Mg^2+^+dox) did not show any alterations in their cell cycle although we did detect a slight increase in cell death in the +Mg^2+^+ dox group (Figure S3).

Taken together, our results indicate that MgtE is able to provide substantial magnesium uptake ability to TRPM7-KO cells, thereby allowing them to grow in regular cell culture media. In addition, MgtE undergoes magnesium-dependent downregulation in a manner akin to its vertebrate homolog, SLC41A1, although this downregulation appears insufficient to maintain normal Mg^2+^ homeostasis as judged by the considerable increase in cell size we observed for MgtE-expressing cells cultured with 15 mM supplemental Mg^2+^.

### TRPM7-KO Cells Expressing the N Domain Deletion Mutant of MgtE Display Stunted Growth

As earlier data from the Δ1–129 MgtE mutant suggested that the cytoplasmic N domain acts as a clamp to hold the dimeric CBS domains in a closed conformation [17], we asked whether the N domain of MgtE would retain similar functionality in TRPM7-KO DT40 cells. We tested this by generating a N-terminal deletion mutant - Δ1–137, and overexpressing it in TRPM7-deficient DT40 cells with an in-frame N-terminal HA-tag. The Δ1–137 mutant is distinct from the Δ1–129 mutant in that it lacks the complete N-terminal domain proximal to the α-helix of the CBS domain [17]. Expression analysis of the HA-tagged Δ1–137 mutant was further confirmed by immunoblotting with anti-HA and revealed a 36 kDa band of expected molecular weight (Figure S1C).

**Figure 5:**
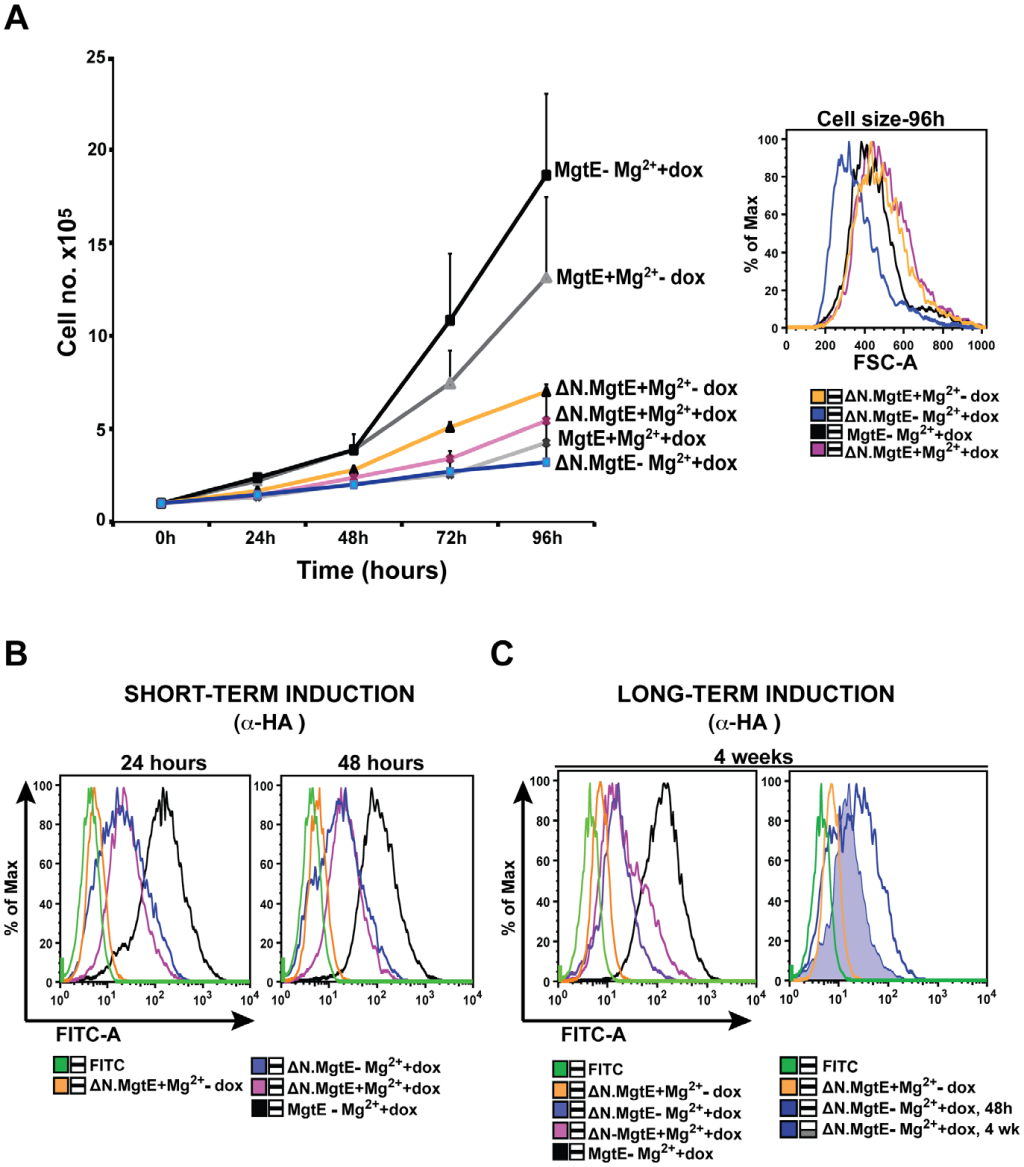
Expression of the N domain deletion mutant results in stunted growth and reduced cell size. (**A**) *Left panel*: TRPM7-KO cells expressing the HA-tagged N domain MgtE deletion (Δ1–137) mutant were either kept uninduced, doxycycline - induced or induced in presence of 15 mM supplemental Mg^2+^ following which their growth was monitored at 24, 48, 72 and 96 hours, respectively. TRPM7-KO cells expressing the HA-tagged WT MgtE were used as controls under similar growth conditions. Growth curves represent the average of three independent experiments and the error bars represent standard error of the mean. *Right panel*: Cell size analysis of TRPM7-KO cells expressing the HA-tagged N domain deletion mutant at 96 hours. Cells were either kept uninduced or induced in absence/presence of 15 mM magnesium and acquired on BD LSRII. Data is representative of at least three independent experiments. (**B**) TRPM7-KO cells expressing the HA-tagged N domain deletion mutant were analyzed for total protein expression by staining with anti-HA and FITC, post fixation and permeabilization. Single stained cells were used as controls and cells were acquired on BD LSRII followed by data analysis using FlowJo software. Comparable expression of the mutant was observed in cells induced in presence/absence of Mg^2+^. Results are representative of three independent experiments. (**C**) TRPM7-KO cells expressing the N domain deletion mutant were maintained under similar conditions as mentioned in (B) and analyzed for total protein expression (anti-HA/FITC) at four weeks. While the cells induced in presence of supplemental Mg^2+^ exhibited slightly increased expression (left panel), cells induced in regular cell culture media for up to 4 weeks displayed significantly lower expression levels compared to the ones induced for only 48 hours (right panel). In both (B) and (C), TRPM7-KO cells expressing the WT MgtE had highest expression at all time points. Results are representative of at least two independent sets of experiments.

Because the Δ1–129 MgtE mutant has a strikingly higher open probability compared to the WT MgtE [17], we hypothesized that expression of the Δ1–137 MgtE deletion mutant would provide growth complementation in TRPM7-KO cells at levels analogous to those observed with the full-length MgtE and additionally, exhibit an even more pronounced downregulation compared to the WT MgtE, in presence of 15 mM supplemental magnesium. However, growth comparison of the mutant with the WT MgtE indicated that TRPM7-KO cells expressing the Δ1–137 mutant displayed significantly stunted growth upon induction while elevated growth levels were observed with cells induced in presence of 15 mM Mg^2+^ (Figure 5A, left panel). Moreover, cell size was commensurate with growth - TRPM7-KO cells expressing the mutant and induced in regular cell culture media were considerably smaller in size than the cells induced in presence of 15 mM supplemental Mg^2+^ as well as cells expressing the WT MgtE, suggesting that the mutant protein is functionally impaired at physiological magnesium concentrations (Figure 5A, right panel). On a side note, we also noted differences in growth between the uninduced TRPM7-KO cells transfected with either the WT MgtE or its mutant (Figure 5A, left panel). This difference in growth could be ascribed in part to clonal variation due to nonspecific selection of the integration site or occurrence of multiple integration events, which could potentially alter expression levels of nearby cellular genes as well as the basal expression level of the transgene. This observed limitation of the tetracycline-regulated expression system has been well documented for other transgenes.

Since we observed a downregulation of WT MgtE in presence of 15 mM Mg^2+^, we examined whether we would observe a similar effect in cells expressing the N domain deletion mutant. In order to ascertain that, we analyzed expression of the deletion mutant in doxycycline-induced TRPM7-KO cells expressing the HA-tagged Δ1–137 mutant, either in absence or presence of 15 mM Mg^2+^ for 24 and 48 hours, by flow cytometry. Interestingly, our analysis revealed comparable expression levels of the Δ1–137 mutant in cells induced either in absence or presence of 15 mM Mg^2+^ at both 24 and 48-hour time points, although the expression of the WT MgtE was overall higher than the mutant at both the time points (Figure 5B). This is in contrast to what we had observed with the wild-type protein and indicates that deletion of the N domain largely abolishes Mg^2+^ dependent downregulation of MgtE.

Similarly, upon prolonged induction for up to 4 weeks, we did not observe any reduction in the expression level of the deletion mutant induced in presence of supplemental Mg^2+^ versus regular cell culture medium, rather cells induced in presence of supplemental Mg^2+^ had somewhat higher expression than the cells induced in regular cell culture media Additionally, cells expressing the WT MgtE still retained an overall high expression at 4 weeks (Figure 5C, left panel). However, we did note a reduction in the expression level of the mutant protein in cells that were induced in absence of Mg^2+^ for prolonged periods versus cells that were induced for only 48 hours (Figure 5C, right panel). These data are again disparate from what we have observed with the WT MgtE and are consistent with the conclusion that the Δ1–137 mutant is functionally impaired and no longer subject to magnesium dependent downregulation.

**Figure 6:**
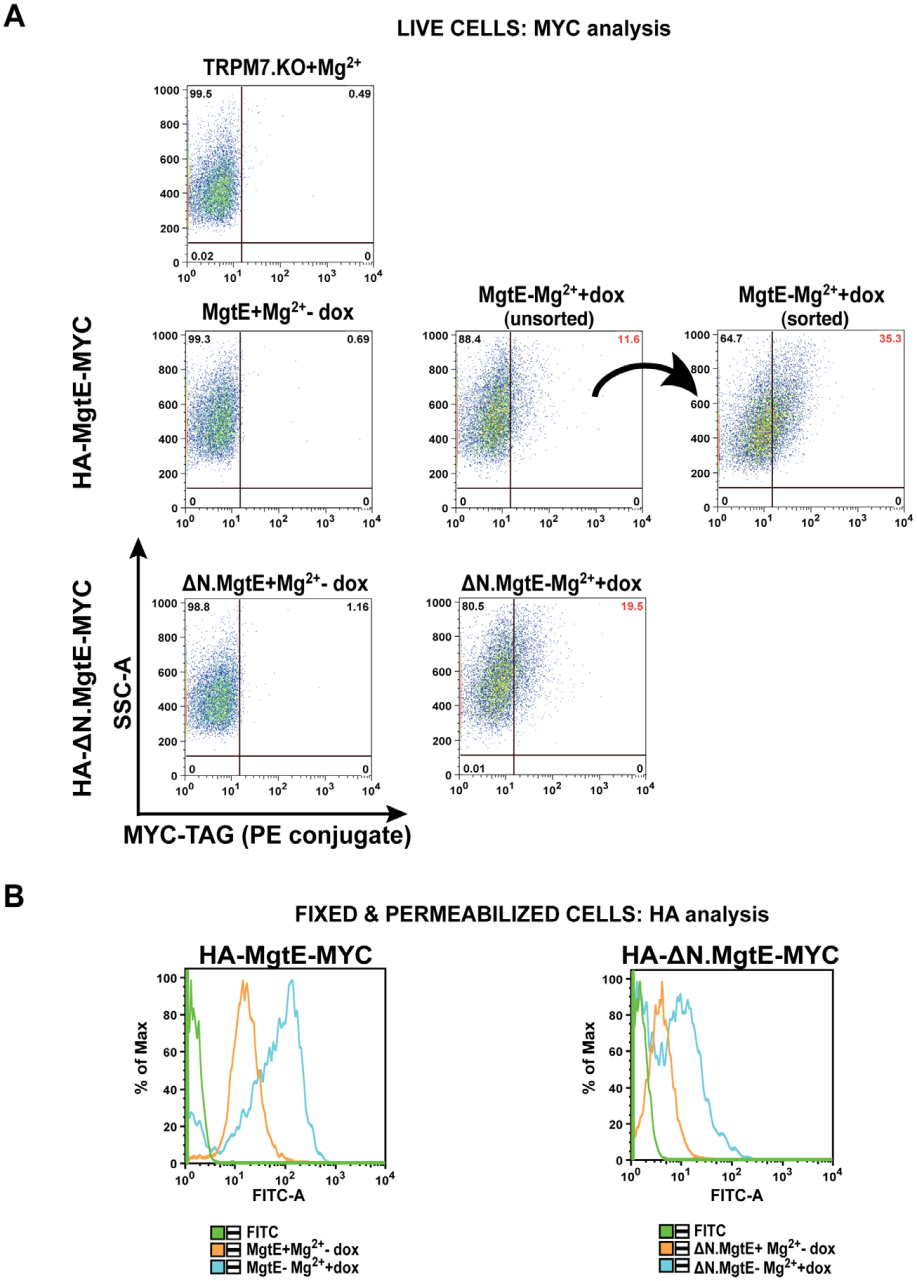
C-terminal MYC-tagged WT MgtE and ΔN domain mutant display surface expression. (**A**) Representative flow plots showing surface expression of the MYC-tagged WT MgtE and the N domain deletion mutant (HA-tagged at the N-terminus and MYC-tagged at the C-terminus) in stably transfected TRPM7-KO cells stained with MYC directly conjugated to PE. While the untransfected TRPM7-KO cells and the uninduced cells did not display any MYC-PE fluorescence, both the WT MgtE and the N domain deletion mutant demonstrated increased mean fluorescence intensities post staining. Sorting and analysis of WT MgtE cells based on the surface expression of MYC showed a significant shift to higher fluorescence intensity. (**B**) Uninduced and doxycycline induced cells in (A) were fixed & permeabilized and probed with anti-HA/anti-mouse FITC for analysis of the N-terminal HA-tag. A strong shift in fluorescence was observed in both induced WT MgtE and N-terminal mutant expressing cells relative to the uninduced controls. A small change in the fluorescence intensity of the uninduced cells was also observed in both the cases indicating a leaky promoter. Plots are representative of at least two independent experiments.

Defective folding and processing of mutated versions of membrane proteins have previously been shown to impair their surface expression as well as affect their function [25], [26]. In order to further test whether the stunted growth displayed by TRPM7-KO cells expressing the Δ1–137 mutant might be due to misfolding and impaired surface trafficking of the protein, we generated surface detectable C-terminal MYC-tagged versions of both the WT and Δ1–137 mutant, which retained the HA-tag at the N-terminus. The double-tagged constructs were stably transfected in DT40 TRPM7-KO cells and analyzed for surface expression of MYC by flow cytometry. As can be seen in Figure 6A, we detected distinct, comparable fluorescence shifts in TRPM7-KO cells inducibly expressing either the MYC-tagged WT or N domain MgtE mutant whereas in contrast, no significant changes in fluorescence were detected in either non-transfected TRPM7-KO or uninduced cells. Furthermore, flow sorting of cells expressing the WT MgtE based on surface MYC expression and their analysis 5 days post-sort, demonstrated further augmentation of the MYC-PE fluorescence, indicative of increased expression of the protein at the plasma membrane (Figure 6A, extreme right panel next to the arrow). Additionally, the intracellular HA-tag was also readily detectable in the fixed and permeabilized cells expressing the double-tagged WT MgtE or the N domain mutant (Figure 6B).

Cumulatively, our data suggests that Δ1–137 truncation of the cytoplasmic N domain of MgtE affects its channel activity but not its surface expression leading to significantly stunted growth secondary to deficient Mg^2+^ uptake.

## Discussion

Given the recent emergence of data with other vertebrate magnesium transporters [5], [6], [7], [10], we speculated that an orthologous Mg^2+^ transporter, MgtE, could functionally compensate for the absence of TRPM7. We chose to evaluate *B. subtilis* MgtE specifically based on its homology with the SLC41 family of vertebrate transporters, of which two members have been characterized in-depth [5], [6], [14], [18]. In this report, we demonstrate for the first time that *B. subtilis* MgtE is able to support the proliferation of vertebrate DT40 B-cells lacking TRPM7, at levels comparable to those of its vertebrate homolog, SLC41A1. Our findings show that MgtE is able to function as a plasma membrane Mg^2+^ transporter in vertebrate cells and highlights the functional similarities between MgtE and SLC41A1. Heterologous expression of the WT or N domain mutant of MgtE in TRPM7-deficient vertebrate cells allowed us to further delineate the role of the N-terminal domain of *B. subtilis* MgtE in Mg^2+^ dependent regulation as well as maintenance of channel function.

Despite the fact that MgtE provides growth complementation to TRPM7-KO cells, we were unable to observe any MgtE specific currents under our experimental conditions. A few possibilities could account for the observed differences between our data and what has been reported earlier [17]. One, it’s conceivable that the patch clamp experiments by Hattori, et al carried out using solutions with a high concentration of sucrose, could potentially influence the permeation characteristics of the channel by altering osmolarity. Second, the different expression systems employed in the studies - bacterial spheroplasts versus DT40 vertebrate cell line could also account for the absence of MgtE-specific currents in our cells. More specifically, as can be seen in our immunofluorescence data, MgtE is predominantly localized in the intracellular compartments in DT40 cells and surface expression levels could be lower than those detected in spheroplasts, leading to smaller currents that are undetectable under physiologic conditions. An important point to consider here is that variability in patch clamp data has also been previously reported in context of both the vertebrate homologs of MgtE - SLC41A1 and SLC41A2, wherein protein specific currents were observed in *Xenopus* oocytes but not in vertebrate expression systems [6], [14], [27], [28]. Resolving the variation between the behavior of MgtE observed in the vertebrate system versus that observed in the spheroplast expression system will be important for an in-depth understanding of its regulatory and transport mechanisms.

Our biochemical analyses pointed towards significantly reduced expression of MgtE after five days of culture in presence of supplemental Mg^2+^ and an even further downregulation of total protein expression after four weeks of continuous culture in presence of 15 mM Mg^2+^. In *B.subtilis,* MgtE is subject to a riboswitch-mediated transcription attenuation dependent on intracellular Mg^2+^ levels [29]. However, the MgtE construct used in our study lacks the 5′UTR that contains the riboswitch and therefore is not subject to this regulatory mechanism. Since the parent cell line that lacks TRPM7 is able to proliferate in presence of supplemental Mg^2+^, its not inconceivable that over time silencing of MgtE could result in lower expression levels without any effect on the proliferative capacity of TRPM7-deficient cells, as shown in Figure 4B, right panel. Moreover, a negative selection mechanism might be at work as essentially MgtE only adopts a closed conformation at concentrations far above the physiological intracellular Mg^2+^ concentration of 2 mM. Since MgtE is unlikely to be subject to regulatory inputs that would normally regulate the opening state of eukaryotic magnesium channels, high expression levels at the plasma membrane and intracellular membranes might in fact be detrimental to growth, which would lead to clonal selection of low expressers. Interestingly, this line of reasoning also hints at the possibility that TRPM7-KO cells might lack an Mg^2+^ efflux mechanism that could compensate for this negative selection pressure.

Hattori and colleagues also demonstrated that the Δ1–129 MgtE deletion mutant exhibits a higher probability to adopt an open conformation [17]. While the function of the N domain is not entirely clear, Hattori et al speculate that the N domain acts as a clamp to secure the CBS domain in the closed state. However, our findings do not fully support this hypothesis. We observed that TRPM7-KO cells expressing the inducible Δ1–137 mutant displayed attenuated growth levels as well as a reduced cell size, reminiscent of quiescent TRPM7-KO cells [30], compared to cells expressing the WT MgtE. This phenotype would be consistent with a hyperfunctioning of the channel at low magnesium concentration if loss of the N domain indeed shifted the equilibrium towards a more open conformation. However, the fact that high concentrations of magnesium restore cell growth as well as cell size and that the mutant is downregulated to a much lower extent than the WT protein at high extracellular magnesium concentrations is contrary to what would be expected of a hyperfunctioning channel. Unless there is an additional mechanism to achieve channel closure in the absence of the N domain, our data appears to suggest that the N domain is in fact necessary for the channel to adopt an open conformation and that loss of this domain forces the TM domain of MgtE into a closed conformation even at low concentrations of magnesium. This could be either due to a gross change in folding of the pore region or stabilization of the plug helices. One possible explanation for the disparity between our observations and Hattori et al’s data could be that our Δ1–137 mutant (made based upon NCBI annotation) is slightly shorter than the Δ1–129 mutant, and lacks an additional residue (G) supposedly involved in Mg^2+^ coordination [17]. Interestingly, despite the functional impairment of Δ1–137 mutant, our data shows that the mutant displays normal protein trafficking and surface expression, which is comparable to the WT MgtE. Taken together, our findings suggests that the N-terminal domains of SLC41A1 and MgtE differ with respect to the regulation of channel activity while both proteins share specificity for magnesium ions. Further studies will be required to elucidate the precise function of the N domain of MgtE.

Besides CorA and MgtA/B, MgtE is one of the three well-characterized bacterial magnesium transporters. It is well established that besides regulating Mg^2+^ uptake under different conditions [2], each transporter is involved in other important functional roles - bacterial adherence [31], virulence [32]–[36], and/or enhanced survival at high temperature [37], processes that are an integral part of bacterial physiology, thereby effectively making them nonredundant. It’s tempting to speculate on broader implications of this finding in context of vertebrate Mg^2+^ transporters, in which each transporter could be involved in mediating Mg^2+^ uptake in response to specific cues, potentially activating the transporter-specific/shared signaling cascade and downstream metabolic pathways. This seems plausible considering that a recent study has shown that mutations in the magnesium transporter, *MAGT1*, results in defective T-cell activation and is characterized by a novel X-linked human immunodeficiency [10]. Ascribing a specific role to each of these vertebrate magnesium transporters in maintenance of Mg^2+^ homeostasis however, remains a challenge and is a goal of our future studies.

## Materials and Methods

### Cell Lines

The DT40 cell line was obtained from Dr. Tomohiro Kurosaki [38] and DT40 TRPM7-deficient/KO cells have been previously described [4]. Both DT40 and TRPM7-KO DT40 cells were maintained in RPMI 1640 with 10% fetal bovine serum (FBS) and 1% chicken serum, 2 mM L-glutamine, and 10 units/ml penicillin/streptomycin at 37°C in a humidified 5% CO_2_ incubator.

### Cloning, Expression and Analysis of MgtE in DT40 B-cells

*Bacillus subtilis* MgtE cDNA was a kind gift from Dr. Michael Maguire at the Case Western Reserve University, Cleveland, OH. The cDNA was transformed and amplified to obtain a DNA prep followed by PCR amplification. The PCR cycle used was: 94°C for 2 min, followed by 34 cycles of 94°C for 30 s, 62°C for 40 s, 72°C for 3 min and a final extension at 72°C for 5 min. The coding sequence was fused at the 5′-end to a Kozak sequence followed by a sequence encoding the HA epitope tag and an additional in-frame NotI site GCGGCCGC. At the 3′-end an XbaI site was added downstream of the stop codon. The PCR product encoded for a modified protein starting with the amino acid sequence MYPYDVPDYAGRPL followed by the MgtE coding sequence and was cloned into the pcDNA5/TO vector (Invitrogen), which provides a tetracycline-controlled expression from a CMV promoter. The pcDNA5/TO-HA-MgtE construct was transfected by electroporation into TRPM7-KO DT40 cells expressing the tet repressor protein (via previous stable transfection with pCDNA6/TR), and clones were selected with Hygromycin (2 mg/ml). The resistant clones were analyzed for tetracycline-induced HA-MgtE expression by immunoblotting and immunoprecipitation. The Δ1–137 N–domain deletion mutant was constructed in a similar manner with an N-terminal HA-tag and stable clones of TRPM7-KO cells expressing the mutant were selected with Hygromycin. Protein expression was further confirmed by immunoblotting. C-terminal MYC-tagged versions of both WT and N-terminal deletion mutant were put together by PCR amplification of the HA-tagged constructs and cloned into pCDNA5/TO. Comparison of the growth rates between the TRPM7-KO cells, TRPM7-KO cells expressing HA-MgtE and TRPM7-KO cells expressing the N-domain deleted HA-MgtE was performed by seeding the cells at an initial cell density of 2 10^5^/ml, following which they were counted at 24, 48, 72 and/or 98 hrs respectively.

### Indirect Immunofluorescence

Both uninduced and doxycycline induced DT40 cells were allowed to attach to glass coverslips in serum free media for 10 mins. Cells were washed once with PBS and fixed with 4% paraformaldehyde for 15 mins at room temperature, permeabilized with 0.1% Triton X-100 and incubated with anti-HA (Cat# 3724; Cell Signaling) at 1∶1000 according to manufacturer’s instructions. After 4 washes with PBS, cells were incubated with the anti-rabbit FITC secondary antibody (Southern Biotech). Post three washes with PBS, a drop of Vectashield mounting medium was added onto each slide and a coverslip was applied. Excess fluid was removed with a paper towel and the coverslips were sealed with transparent nail polish and dried. Images were captured by fluorescence microscope (Leica DM6000B, Leica Microsystems) equipped with a digital camera.

### Electrophoresis, Immunoprecipitations and Western Blotting

Sodium dodecyl sulfate-polyacrylamide gel electrophoresis (SDS-PAGE) was carried out according to the method of Laemmli using 10% gels. Whole cell lysates were prepared by lysing the cells in ice-cold lysis buffer (150 mM NaCl, 50 mM Tris pH 7.5, 1% Triton X-100 or Nonidet P-40 supplemented with protease inhibitors). The lysate was rotated for 45 min at 4°C followed by centrifugation at 20,000 g for 15 mins and protein concentration was determined by the BCA assay kit using the manufacturer’s instructions (Pierce). Anti-HA (HA-Tag Mouse mAb; Cell Signaling) immunoprecipitations were performed on pre-cleared cell lysates of DT40 cells at 4°C by addition of protein A-Sepharose beads. Subsequently, the beads were washed three times with lysis buffer, resuspended in sample loading buffer and boiled for 6–7 mins. Aliquots of the supernatant were separated on 10% SDS-PAGE gels. Proteins were transferred to PVDF membrane (Millipore) and were blocked in 5% blocking buffer for 1 hr at room temperature (5% w/v nonfat dry milk in TBS-0.1%Tween-20) followed by overnight incubation in the primary antibody. Bound antibody was detected by the ECL chemiluminescence detection system the next day (Amersham).

### Membrane Topology Analysis by Flow Cytometry

TRPM7-KO DT40 B-cells (2×10^5^–5×10^5^) expressing either HA-tagged MgtE or the HA-tagged Δ1–137 mutant were harvested and washed with PBS. For surface staining, cells were washed twice with PBS+1% BSA and stained with respective anti-HA and secondary antibodies. For detection of intracellular epitopes, cells were fixed and permeabilized with BD cytoperm/cytofix solution (Cat# 554714) at 4°C for 20 min, prior to incubation with the primary antibody. Next, cells were stained with the primary antibody (α-HA; Cell Signaling, Cat# 2367) at 4°C for 30 mins and washed twice with PBS +1% BSA. Fixed and permeabilized cells were washed with the BD perm/wash solution (1×). Further, cells were incubated with species-specific FITC secondary antibody at 4°C for 25 mins and washed twice. Cells were resuspended in PBS+1% BSA and analyzed on BD LSRII. For surface detection of MYC, double-tagged WT and N-domain deletion mutant (HA at N-terminus and MYC at C-terminus) were stably transfected into DT40 TRPM7-KO cells. HA-tag expression was analyzed and verified by flow cytometry and clones were subsequently checked for surface expression of MYC-tag with anti-MYC directly conjugated to PE (Cell Signaling, Cat#3739) and acquired on BD LSRII.

### Electrophysiology

WT and TRPM7-KO DT40 B-cells were previously described [4]. TRPM7-KO clone expressing HA-MgtE (stably transfected with pcDNA5-TO/HA-MgtE construct driving tetracycline-inducible MgtE expression) was constructed as above. Expression of MgtE was induced by adding 1 µg/ml doxycycline to the culture medium.

Whole cell patch clamp experiments were performed at 21–25°C. Cells that were patched approximately 2–4 hours after plating on glass coverslips were kept in a standard modified Ringer’s solution of the following composition (in mM): NaCl 145, KCl 2.8, CsCl 10, CaCl_2_ 1, MgCl_2_ 2, glucose 10, Hepes·NaOH 10, pH 7.2. Nominal zero intracellular pipette-filling solutions contained (in mM): Cs-glutamate 145, NaCl 8, Cs-EGTA 10, Hepes·CsOH 10, pH 7.2. High-resolution current recordings were acquired by a computer-based patch-clamp amplifier system (EPC-10, HEKA, Lambrecht, Germany). Immediately following establishment of the whole-cell configuration, voltage ramps of 50 ms duration spanning the voltage range of –100 to +100 mV were delivered from a holding potential of 0 mV at a rate of 0.5 Hz over a period of 300 to 400 seconds. All voltages were corrected for a liquid junction potential of 10 mV between external and internal solutions when internal solutions contained glutamate. Currents were filtered at 2.3 kHz and digitized at 100 µs intervals. Capacitive currents and series resistance were determined and corrected before each voltage ramp using the automatic capacitance compensation of the EPC-10. The low-resolution temporal development of currents at a given potential was extracted from individual ramp current records by measuring the current amplitudes at voltages of −80 mV or +80 mV. Outward currents are reported for presentation purposes because of their larger magnitude relative to background and direct correlation with TRPM7 activation.

## Supporting Information

Figure S1 **MgtE is expressed in high abundance and displays heat-induced aggregation.** (**A**) Immunoblot analysis of uninduced and doxycycline-induced TRPM7-KO cells expressing HA-tagged MgtE with anti-HA. (**B**) *Left panel*: Cell lysates from doxycycline-induced cells expressing HA-tagged MgtE were either left unheated or heated for 5 minutes and loaded on SDS-PAGE, immunoblotted and probed with anti-HA. *Right panel*: HA-tagged SLC41A1 was treated in a manner similar to the HA-tagged MgtE and immunoblotted followed by staining with anti-HA. (**C**) Full-length HA-MgtE was heated/unheated and immunoblotted along with heated/unheated HA-tagged Δ1−137 N-terminal deletion mutant.
(TIF)

Figure S2 **Mg^2+^ mediated downregulation is specific to MgtE.** (**A**) Flow plots showing total protein expression of human pyruvate kinase M2 (PKM2), HA-tagged at the N-terminus and stably expressed by the DT40 WT cells. Cells were fixed & permeabilized, harvested at 24 h and 48 h post doxycycline induction and analyzed for total HA expression on BD LSRII. No significant fluorescence shifts were observed at either 24 or 48 hours in cells induced in cell culture media with or without 15 mM supplemental Mg^2+^. (**B**) Fixed & permeabilized DT40 TRPM7-KO cells inducibly expressing FLAG - (N-terminus) and HA (C-terminus)-tagged human SLC41A2 were analyzed for total protein expression by staining with anti-HA/FITC. The left and middle panels show analysis of cells induced at 24 and 48 hours in media with or without supplemental Mg^2+^. The right panel shows cells that had been induced for at least three weeks before addition of 15 mM Mg^2+^ for 24 hours. No overall change in fluorescence was detected in cells induced for either short or long term in media with or without supplemental Mg^2+^. All data are representative of two independent experiments.
(TIF)

Figure S3 **Cell cycle and percent live cell analysis of TRPM7-KO cells expressing WT MgtE.** (**A**) DNA content analysis of cell cycle distribution of TRPM7-KO cells stably expressing HA-tagged WT MgtE. Cells were kept either uninduced (+Mg^2+^- dox; left panel), induced (-Mg^2+^+ dox; middle panel) or induced in presence of 15 mM supplemental Mg^2+^ (+Mg^2+^+ dox; right panel) for a period of 96 hours and fixed, followed by staining with DAPI. No significant differences were observed in G1, S or G2 phases under any condition. Results are representative of two independent experiments. (**B**) Forward scatter (FSC-A) versus side scatter (SSC-A) of cells from the same group as above (A) at 96 hours but before fixation. Both uninduced and induced cells had more live cells (gated and in red) than the ones induced in presence of 15 mM Mg^2+^. Results are representative of three independent experiments.
(TIF)
