# Supplementary figures and images for: The B. subtilis MgtE Magnesium Transporter Can Functionally Compensate TRPM7-Deficiency in Vertebrate B-Cells
Source: PLoS One. 2012 Sep 6;7(9):e44452. doi: 10.1371/journal.pone.0044452 (PMC3435302; doi:10.1371/journal.pone.0044452)

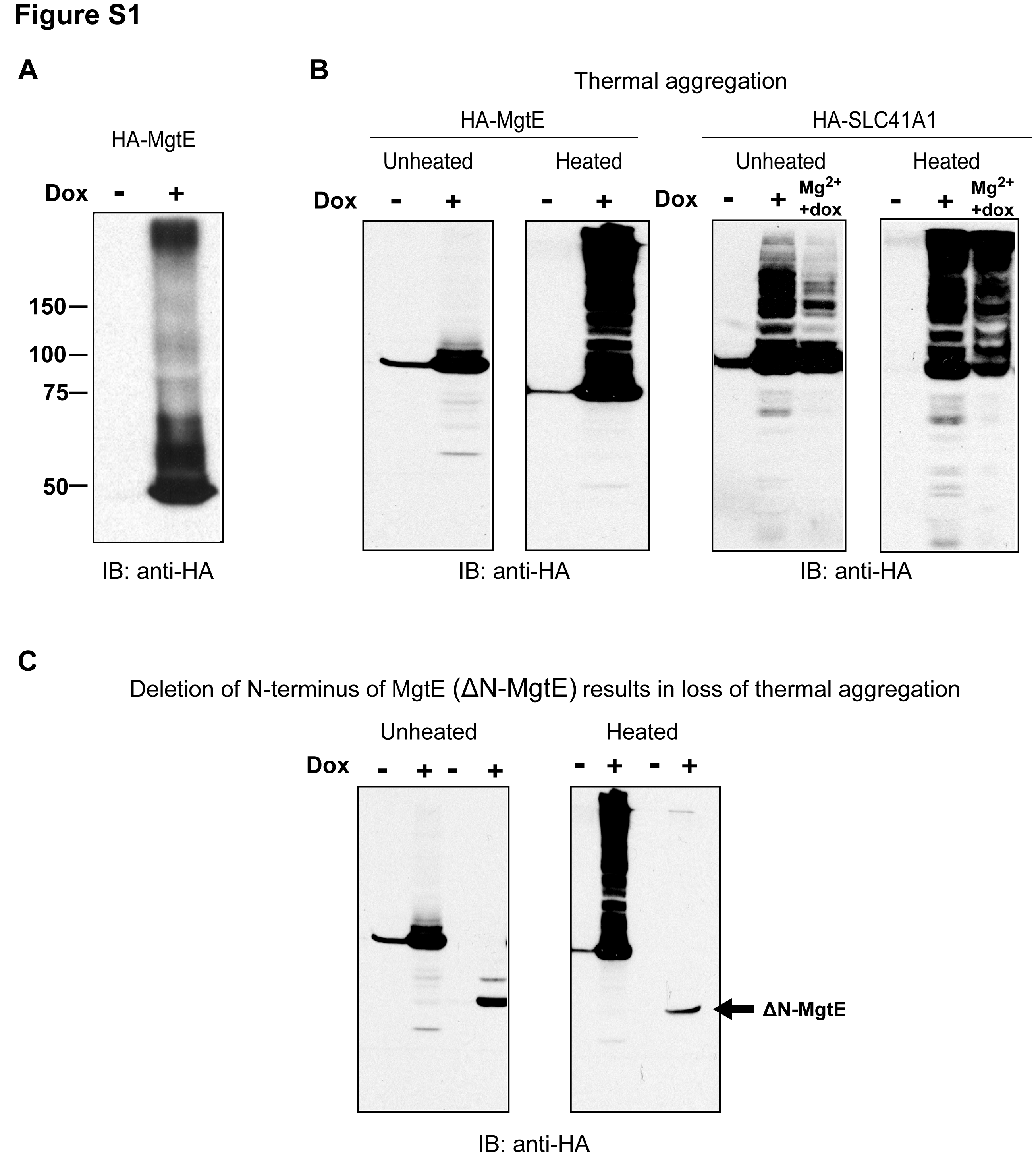

Supplement: Figure S1 — MgtE is expressed in high abundance and displays heat-induced aggregation. (A) Immunoblot analysis of uninduced and doxycycline-induced TRPM7-KO cells expressing HA-tagged MgtE with anti-HA. (B) Left panel: Cell lysates from doxycycline-induced cells expressing HA-tagged MgtE were either left unheated or heated for 5 minutes and loaded on SDS-PAGE, immunoblotted and probed with anti-HA. Right panel: HA-tagged SLC41A1 was treated in a manner similar to the HA-tagged MgtE and immunoblotted followed by staining with anti-HA. (C) Full-length HA-MgtE was heated/unheated and immunoblotted along with heated/unheated HA-tagged Δ1−137 N-terminal deletion mutant. (TIF) [file pone.0044452.s001.tif]

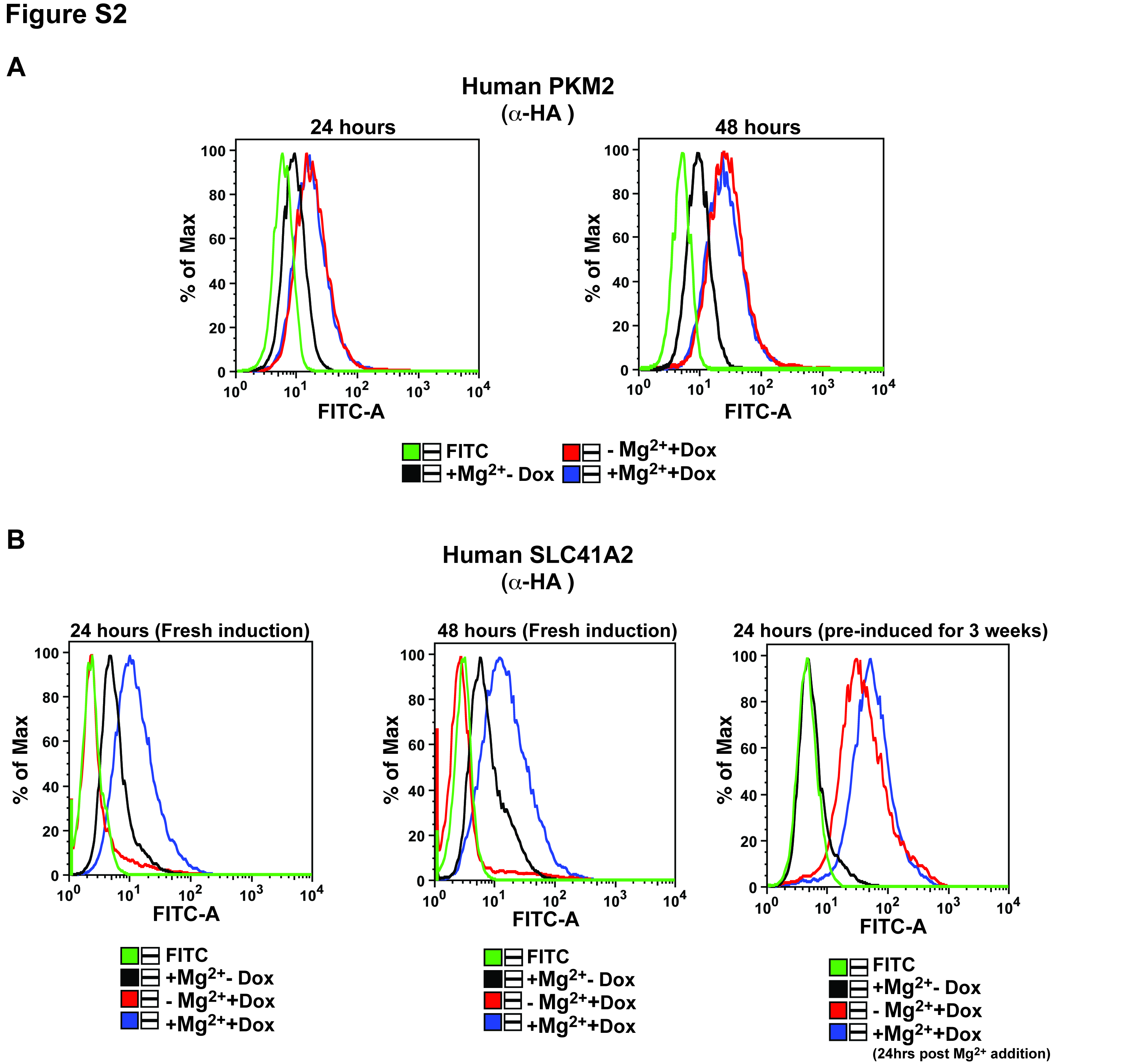

Supplement: Figure S2 — Mg2+ mediated downregulation is specific to MgtE. (A) Flow plots showing total protein expression of human pyruvate kinase M2 (PKM2), HA-tagged at the N-terminus and stably expressed by the DT40 WT cells. Cells were fixed & permeabilized, harvested at 24 h and 48 h post doxycycline induction and analyzed for total HA expression on BD LSRII. No significant fluorescence shifts were observed at either 24 or 48 hours in cells induced in cell culture media with or without 15 mM supplemental Mg2+. (B) Fixed & permeabilized DT40 TRPM7-KO cells inducibly expressing FLAG - (N-terminus) and HA (C-terminus)-tagged human SLC41A2 were analyzed for total protein expression by staining with anti-HA/FITC. The left and middle panels show analysis of cells induced at 24 and 48 hours in media with or without supplemental Mg2+. The right panel shows cells that had been induced for at least three weeks before addition of 15 mM Mg2+ for 24 hours. No overall change in fluorescence was detected in cells induced for either short or long term in media with or without supplemental Mg2+. All data are representative of two independent experiments. (TIF) [file pone.0044452.s002.tif]

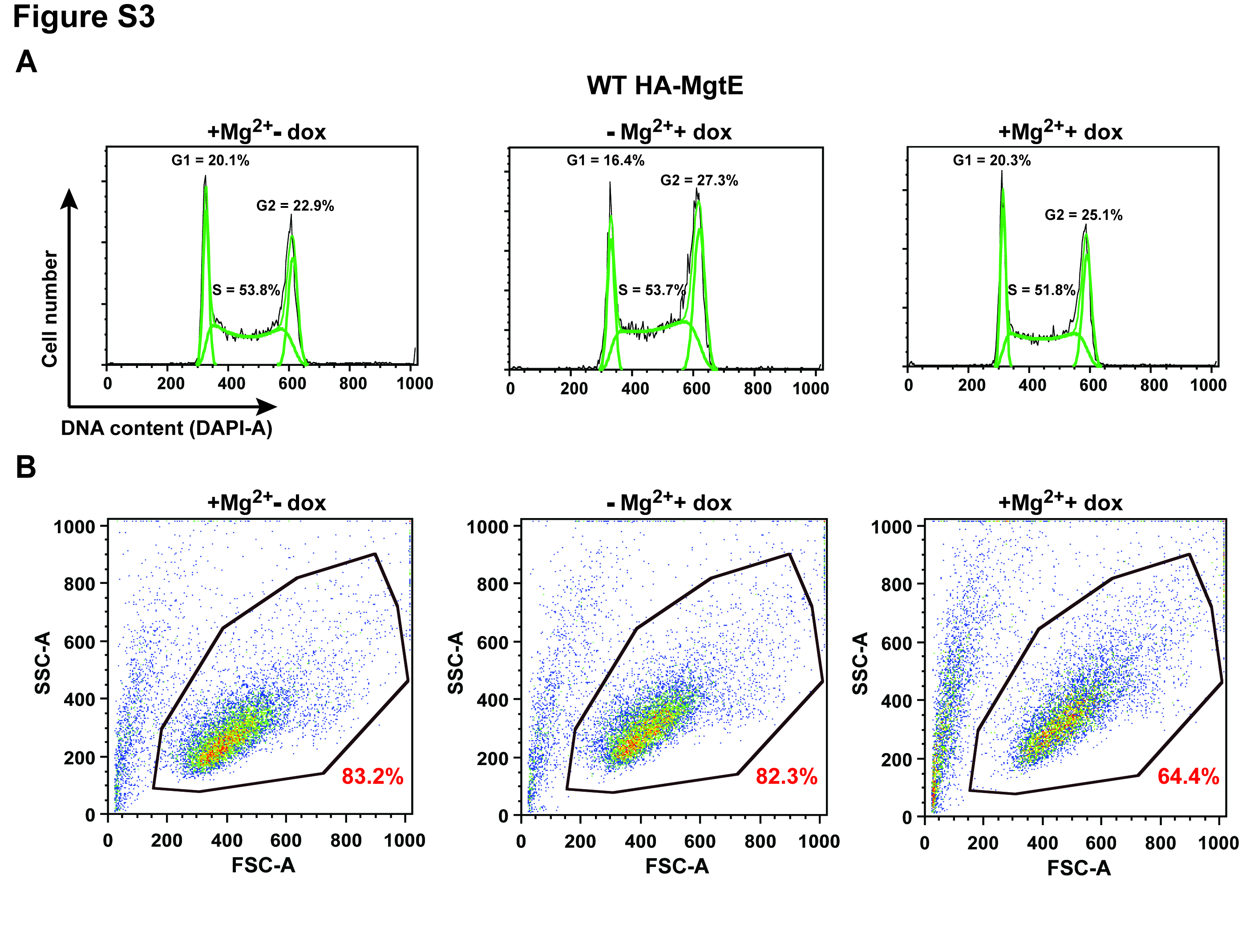

Supplement: Figure S3 — Cell cycle and percent live cell analysis of TRPM7-KO cells expressing WT MgtE. (A) DNA content analysis of cell cycle distribution of TRPM7-KO cells stably expressing HA-tagged WT MgtE. Cells were kept either uninduced (+Mg2+- dox; left panel), induced (-Mg2++ dox; middle panel) or induced in presence of 15 mM supplemental Mg2+ (+Mg2++ dox; right panel) for a period of 96 hours and fixed, followed by staining with DAPI. No significant differences were observed in G1, S or G2 phases under any condition. Results are representative of two independent experiments. (B) Forward scatter (FSC-A) versus side scatter (SSC-A) of cells from the same group as above (A) at 96 hours but before fixation. Both uninduced and induced cells had more live cells (gated and in red) than the ones induced in presence of 15 mM Mg2+. Results are representative of three independent experiments. (TIF) [file pone.0044452.s003.tif]
